# Ferroptosis-induced oxidative stress in therapy-resistant glioblastoma

**DOI:** 10.1038/s41420-026-03227-3

**Published:** 2026-06-26

**Authors:** Sofia Remedia, Cristina Martelli, Marcella Bonanomi, Bruno Giovanni Galuzzi, Francesca Servidio, Clarissa Gervasoni, Martina Nespoli, Chiara Pellizzer, Alessandro Giammona, Daniela Gaglio, Daniele Capitanio, Gloria Bertoli, Luisa Ottobrini, Alessia Lo Dico

**Affiliations:** 1 Institute of Bioimaging and Biological Complex Systems (IBSBC), National Research Council (CNR), Segrate (MI), Italy; 2 NBFC, National Biodiversity Future Center, 90133 Palermo, Italy; 3 Department of Earth and Marine Sciences (DISTEM), University of Palermo, Via Archirafi, 22, 90123 Palermo, Italy; 4 Department of Pathophysiology and Transplantation, University of Milan, 20054 Segrate (MI), Italy; 5 Department of Biomedical Sciences for Health, University of Milan, 20054 Segrate (MI), Italy

**Keywords:** CNS cancer, Stress signalling

## Abstract

Temozolomide (TMZ) resistance remains a major obstacle in glioma treatment. Ferroptosis, an iron-dependent, lipid peroxidation–driven regulated cell death, represents a promising alternative strategy. We investigated molecular determinants of ferroptosis sensitivity in TMZ-resistant glioma, focusing on HIF-1α and oxidative stress. One TMZ-sensitive (U251) and three TMZ-resistant (T98, U118, LN18) cell lines were treated with ferroptosis inducers (Erastin, FIN56, RSL3). Viability, ROS (total and mitochondrial), lipid peroxidation, and ferroptosis-related gene/protein expression were assessed. MitoTEMPO and deferoxamine (DFX) were used to probe mitochondrial ROS and iron dependence, respectively. Integrated metabolomic–proteomic analyses and an orthotopic U118-Luc model supported in vitro findings. Ferroptosis inducers reduced viability and bypassed TMZ resistance in vitro and suppressed tumor growth in vivo with partial body-weight loss. Resistant cells displayed high HIF-1α with elevated GSH/NRF2 activity and reduced lipid peroxidation. Treatments increased oxidative stress and downregulated *GPX4*, *xCT*, *FSP1*, and *ATF4*; MitoTEMPO rescued cells, implicating mitochondrial ROS. Ferroptosis induction reduced HIF-1α expression/nuclear localization and decreased *HIF-1α*/*VEGF*/*SOX2*/*NRF2* in tumor tissue, supporting therapeutic potential.

## Introduction

Glioblastoma (GBM) is among the most aggressive primary brain tumors, typically classified within the glioma group. Despite advances in treatment, GBM remains one of the deadliest human cancers, with a median survival of approximately 14.6 months and a high incidence of recurrence [1]. A major challenge in its clinical management is the development of chemoresistance mechanisms, which render the tumor refractory to standard therapies and further exacerbate disease progression [2]. Over time, GBM commonly acquires resistance to both chemotherapies, such as temozolomide (TMZ) and radiation therapy [3]. This resistance is multifactorial, involving a complex interplay of molecular, biochemical, and metabolic adaptations that make the tumor particularly difficult to treat effectively [4]. Recent evidence has highlighted the role of oxidative stress in GBM pathophysiology, particularly in relation to therapeutic resistance. GBM cells often exhibit elevated levels of reactive oxygen species (ROS) [5]. While ROS can cause significant damage to cellular components such as DNA, proteins, and lipids, many cancer cells, including those of GBM, adapt to high ROS levels by upregulating antioxidant defense systems [6]. This adaptation enables their survival under otherwise lethal oxidative conditions, which would typically induce cell death in non-transformed cells. In this context, further exacerbation of oxidative stress may represent a promising strategy to selectively induce cytotoxicity in tumor cells [7]. Against this backdrop, ferroptosis has emerged as a novel therapeutic approach with the potential to overcome resistance mechanisms and promote tumor cell death [8]. Ferroptosis is an iron-dependent form of regulated cell death, driven by the dysregulation of redox homeostasis and characterized by the accumulation of lipid peroxides due to compromised antioxidant defenses [9]. This process initiates a cascade of molecular and biochemical alterations that culminate in cell death. It differs from apoptosis, necrosis, autophagy, or pyroptosis because it is driven by the Fenton reaction and glutathione (GSH) depletion, leading to the buildup of reactive oxygen species (ROS). In fact, when iron homeostasis is disrupted, excess iron generates H₂O₂, and lipid peroxides produce ROS via the Fenton reaction, triggering ferroptosis [10]. Recent studies have elucidated a complex relationship between Hypoxia-Inducible Factors (HIFs) signaling and ferroptosis, noting that HIFs may employ dual roles either promoting or inhibiting ferroptosis depending on the cellular context [11]. Understanding how HIFs regulate ferroptosis could reveal new therapeutic opportunities, particularly in the hypoxic microenvironment typical of GBM. In fact, HIFs are known to modulate cellular antioxidant systems, which are central to the suppression of ferroptosis. The glutathione (GSH)/glutathione peroxidase 4 (GPX4) axis is a key antioxidant pathway involved in ferroptosis regulation [2]. HIF-1α, under hypoxic conditions, has been shown to upregulate GPX4 expression, thereby influencing the cell’s capacity to neutralize lipid peroxides and prevent ferroptotic death confer cell protection by attenuating oxidative damage [12]. Additionally, the transcription factor Nuclear factor erythroid 2–related factor 2 (NRF2), activated in response to oxidative stress, plays a pivotal role in regulating the expression of antioxidant genes [13]. Emerging evidence suggests that HIF-1α and NRF2 may act synergistically under hypoxic conditions to enhance antioxidant gene expression and suppress ferroptosis, thus reducing lipid peroxidation and ROS accumulation [14]. In the present study, we investigated the effects of oxidative stress induction via ferroptosis inducers in a panel of human GBM cell lines. We evaluated the efficacy of three ferroptosis-inducing compounds, assessing their impact on cell viability and validating ferroptosis as the underlying mechanism of action. Notably, in TMZ-resistant GBM cell lines characterized by elevated basal ROS levels, these compounds further amplified ROS production, inducing a cascade of molecular, biochemical, and functional events consistent with ferroptotic cell death. Comparatively, the pharmacological inhibition of antioxidant systems through genetic knockdown of xCT or GPX4 was less effective than treatment with the ferroptosis inducers, emphasizing the compounds’ ability to robustly engage cell death mechanisms. Furthermore, the critical role of ROS release confirmed by co-treatment with ROS scavengers, which abrogated the cytotoxic effects of the ferroptosis-inducing drugs. To further validate these findings in a more clinically relevant setting, we employed orthotopic GBM mouse models. Using optical imaging strategies and resistant GBM cell lines, we confirmed the therapeutic efficacy of these agents in vivo. These findings suggest that ferroptosis may serve as a promising strategy for overcoming therapy resistance in gliomas. By targeting iron metabolism, lipid peroxidation, and antioxidant defense systems, ferroptosis-inducing therapies hold the potential to significantly improve treatment outcomes. Future preclinical and clinical investigations will be essential to establish the safety, efficacy, and optimal integration of these therapies into current GBM treatment protocols [15].

## Results

### GPX4 and xCT as central drivers of ferroptosis activation in glioma

**Fig. 1:**
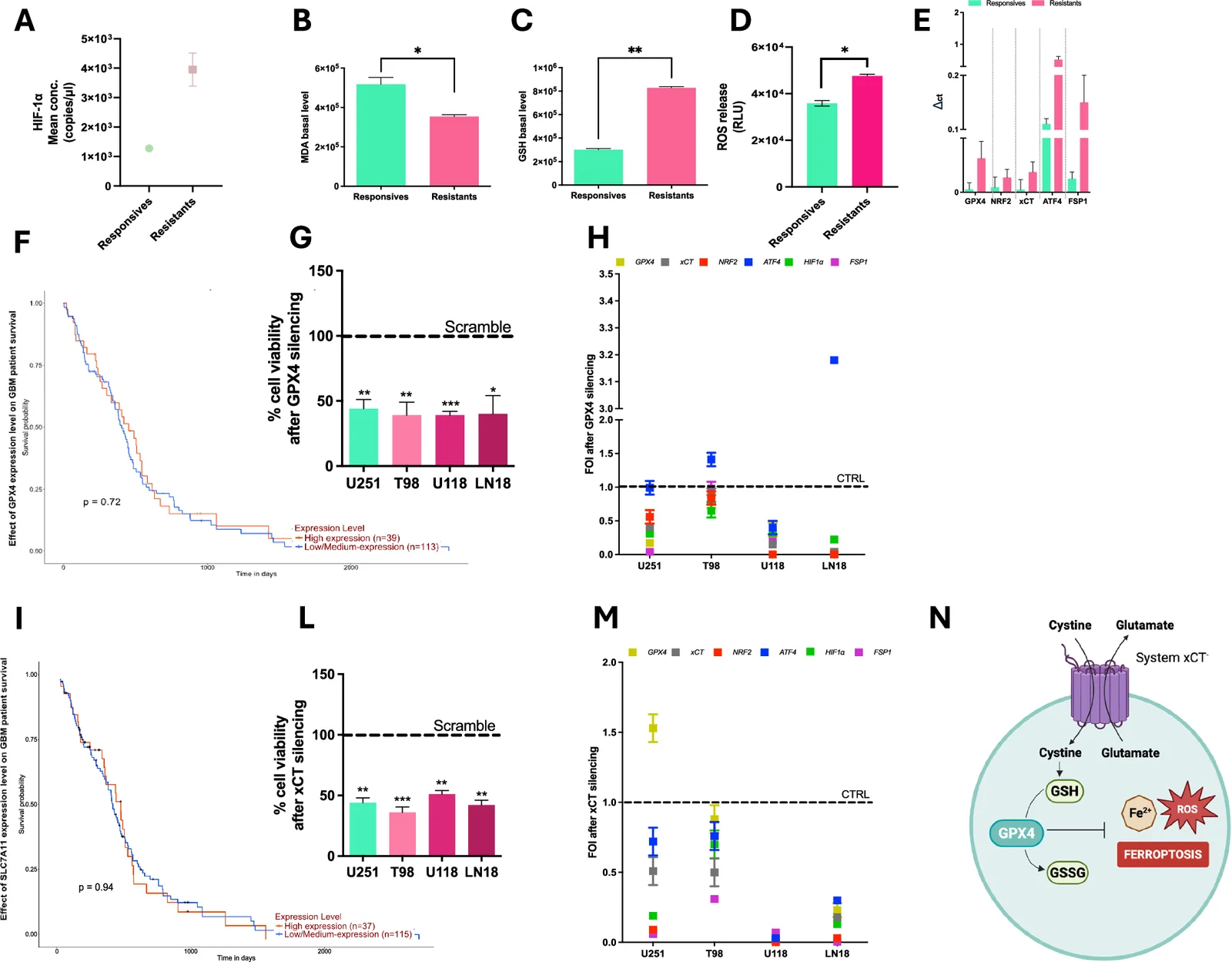
xCT and GPX4 define basal oxidative status and ferroptosis resistance in glioblastoma cells. **A** Basal HIF-1α protein levels in treatment-responsive and -resistant glioblastoma cell lines, quantified by digital PCR as gene copies/µL. **B** Basal lipid peroxidation measured by MDA assay and expressed as fluorescence units (FLU). **C** Intracellular reduced glutathione (GSH) levels are elevated in resistant cells. **D** Total ROS levels assessed by fluorescence-based assay, showing lower oxidative stress in resistant lines. **E** Basal mRNA expression of ferroptosis- and redox- related genes (*SLC7A11*, *GPX4*, *NRF2*, *ATF4*, *FSP1*), upregulated in resistant cells. **F–H** Kaplan–Meier survival analysis of glioblastoma patients stratified by GPX4 expression (UALCAN database) shows no association with overall survival (log-rank test, *p* = 0.72). GPX4 silencing by siRNA reduces cell viability and modulates ferroptosis-related gene expression in resistant cells. **I–M** Kaplan–Meier analysis for SLC7A11 (xCT) expression shows no association with overall survival (log-rank test, *p* = 0.94). xCT silencing reduces cell viability and affects the expression of ferroptosis-related genes in resistant cells. **N** Schematic representation of the xCT–GSH–GPX4 axis: cystine import via xCT sustains GSH synthesis required for GPX4-mediated detoxification of lipid peroxides (LOOH); pathway disruption leads to ROS accumulation and ferroptosis. Data are presented as mean ± SD from at least three independent experiments (*n* = 3), each performed in technical triplicates unless otherwise specified. Quantitative analyses were performed using Real-Time PCR and commercial assays (e.g., GSH, MDA, ROS). Kaplan–Meier survival analyses were generated using the UALCAN database (http://ualcan.path.uab.edu) and curves were analyzed using the log-rank (Mantel-Cox) test. *p* < 0.05 was considered statistically significant.

To characterize the main molecular differences between TMZ-responsive and TMZ-resistant glioma cells, the level of different molecules or pathways were analyzed. Firstly, the analysis reveals that the higher distinguishing feature between the two types of cells is the expression of *HIF-1α*. Absolute quantification of the transcription factor shows a higher number of copies/μL of the gene in resistant cells compared to responsive ones (Fig. 1A). Therefore, TMZ-resistant cells have a lower level of lipid peroxidation (Fig. 1B) associated with higher GSH activity (Fig. 1C). Furthermore, the level of ROS release was found to be higher in resistant cells (Fig. 1D), as well as higher gene expression of genes primarily involved in ferroptosis (Fig. 1E). These findings suggest that HIF-1α may transcriptionally regulate components of the ferroptosis defense machinery in hypoxic or resistant tumor environments. Focusing on two key mediators of ferroptosis, *GPX4* and *xCT*, computational analyses support the hypothesis that low *GPX4* expression correlates with improved survival in Kaplan-Meier curves (Fig. 1F). Supporting this, *GPX4*-silenced resistant cell lines show a significant reduction in cell viability (Fig. 1G). Molecular studies confirm that the ferroptosis-related genes were in line with its activation also by *GPX4* silencing (Fig. 1H). Similarly, elevated *xCT* expression is associated with poorer survival outcomes (Fig. 1I) and in the U118 TMZ-resistant cell line, silenced for *xCT* a statistically significant reduction in cell viability was observed (Fig. 1L). Also, in these silencing experiments, all ferroptosis molecular signatures were in line with its switch off (Fig. 1M). Both findings are consistent with the idea of targeting ferroptosis-related pathways, particularly through inhibition of *xCT* or *GPX4*. Together, these results highlight a critical role for the *HIF-1α/xCT*/*GPX4* axis in modulating ferroptosis resistance in glioma cells (Fig. 1N). The differential expression of these key regulators not only distinguishes TMZ-resistant from TMZ-responsive phenotypes but also suggests potential therapeutic vulnerabilities. From this point onward, we have considered four distinct cell lines: one TMZ-responsive (U251) and three TMZ-resistant (T98, U118 and LN18).

### Pharmacological induction of ferroptosis requires HIF-1α downregulation in glioma models

**Fig. 2:**
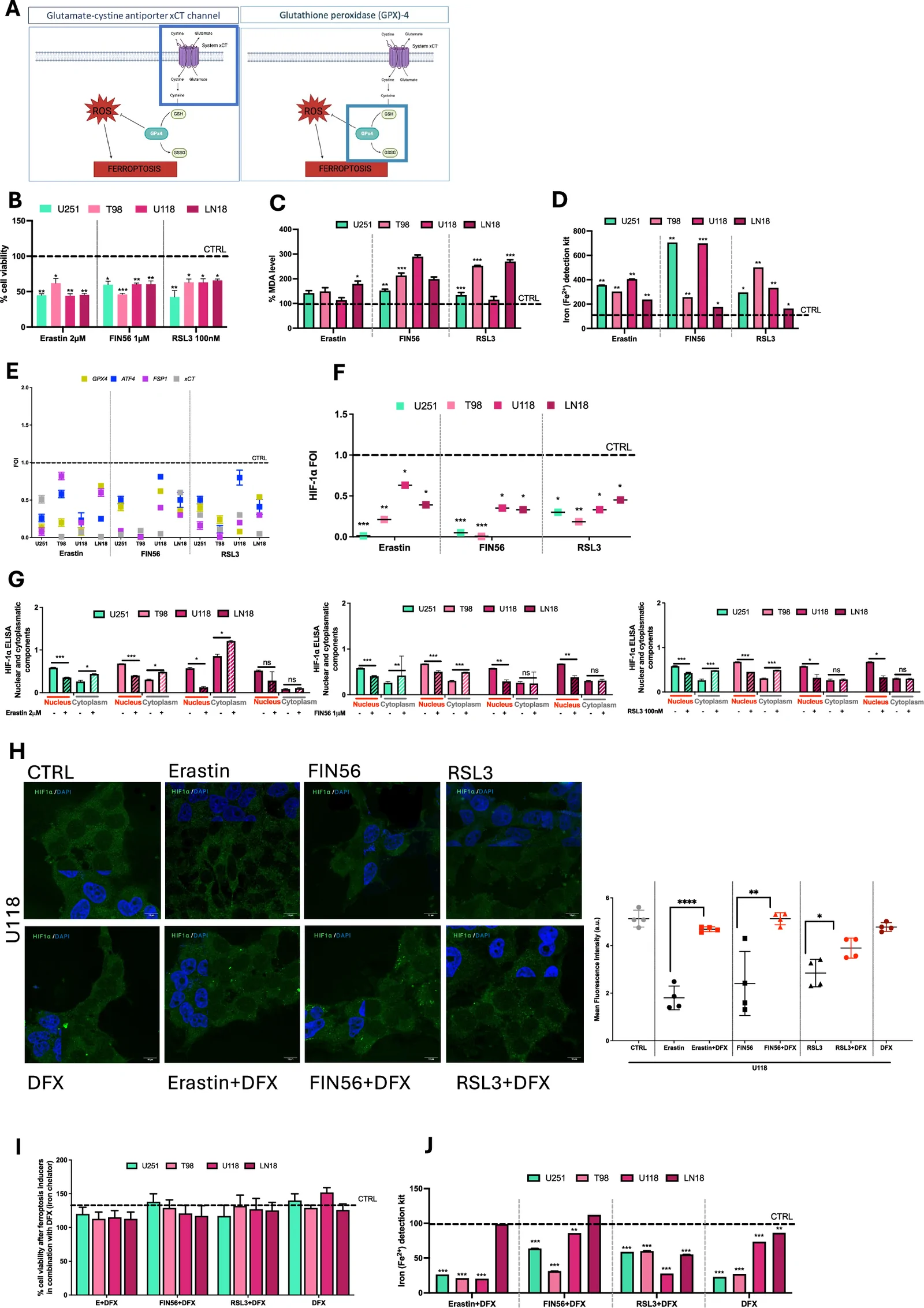
Disruption of HIF-1 signaling enhances ferroptosis susceptibility in glioblastoma via iron accumulation and lipid peroxidation. **A** Schematic of ferroptosis pathways involving xCT (SLC7A11) and GPX4 in ROS detoxification and cell survival. **B** Cell viability in three resistant and one responsive glioblastoma cell lines following treatment with Erastin, FIN56, or RSL3. **C** Lipid peroxidation levels (MDA assay) after drug exposure in the same cell lines. **D** Intracellular iron accumulation measured after treatment with ferroptosis inducers. **E** Expression of ferroptosis-related genes (e.g., *GPX4*, *SLC7A11*, *FSP1*) following treatment. **F** mRNA expression levels of HIF-1α following ferroptosis induction. **G** HIF-1α nuclear and cytoplasmic protein levels quantified by ELISA after treatment, with deferoxamine (DFX) used as positive control. **H** Confocal immunofluorescence analysis of HIF-1α subcellular localization in glioblastoma cells treated with ferroptosis inducers or in combination with DFX. Effect of iron chelation on ferroptosis: cell viability (**I**) and intracellular iron levels (**J**) after co-treatment with ferroptosis inducers and DFX. Data are presented as mean ± SD from at least three independent biological experiments (*n* = 3), each performed in technical triplicates unless otherwise specified. Statistical analyses were performed using one-way ANOVA followed by appropriate post hoc tests for multiple comparisons, or unpaired two-tailed Student’s *t* test for pairwise comparisons, using GraphPad Prism 11. A *p* value < 0.05 was considered statistically significant (*p < 0.05, **p < 0.01, ***p < 0.001).

The actual contribution of ferroptosis was therefore explored using three ferroptosis-inducing drugs, whose mechanisms and targets are schematically visible in Fig. 2A. Following a preliminary dose-response study [Supplementary 1], the efficacy of the drugs in reducing their direct targets at both the genetic and functional levels was validated [Supplementary 2]. Therefore, the identified dose was applied in cell viability assays across the full panel of cell lines, confirming the effectiveness of the three drugs in reducing cell viability (Fig. 2B). Subsequently, the activation of ferroptosis was investigated following treatment, revealing an increase in lipid peroxidation (Fig. 2C and Supplementary 3) and intracellular iron concentration (Fig. 2D) both consistent with a gene expression profile associated with ferroptosis specific pathway. Specifically, all key ferroptosis-related genes, including *xCT*, *NRF2*, *GPX4*, *ATF4*, and *FSP1*, were found to be downregulated (Fig. 2E). Returning to the HIF-1α analysis, all treatments that induce ferroptosis were found to reduce *HIF-1α* at the molecular level (Fig. 2F). Importantly, also its activity was reduced in parallel with decreased nuclear localization, as confirmed by immunofluorescence analysis, demonstrating changes at both the functional and visual levels (Fig. 2G, H) [16]. The role of HIF-1α in ferroptosis was further confirmed by combined treatment with the hypoxia-mimetic agent, Deferoxamine (DFX), which increases the nuclear accumulation of the biologically active HIF-1α complex. Under normoxic conditions, the HIF-1α subunit is predominantly localized in the cytoplasm, where it is rapidly degraded. However, DFX stabilizes HIF-1α, allowing it to translocate into the nucleus and form the active *HIF-1* transcription factor (Fig. 2H) [17]. This nuclear activation counteracted the effects of the three ferroptosis-inducing drugs impairing the reduction of cell viability (Fig. 2I), suggesting that effective induction of ferroptosis also requires suppression of HIF-1α activity. Notably, co-treatment shifted the iron accumulation mandatory for ferroptosis switch on allowing the cells to shift their profile toward inhibition of the process (Fig. 2J).

### Mitochondrial oxidative stress and NRF2 suppression cooperate in ferroptosis induction

**Fig. 3:**
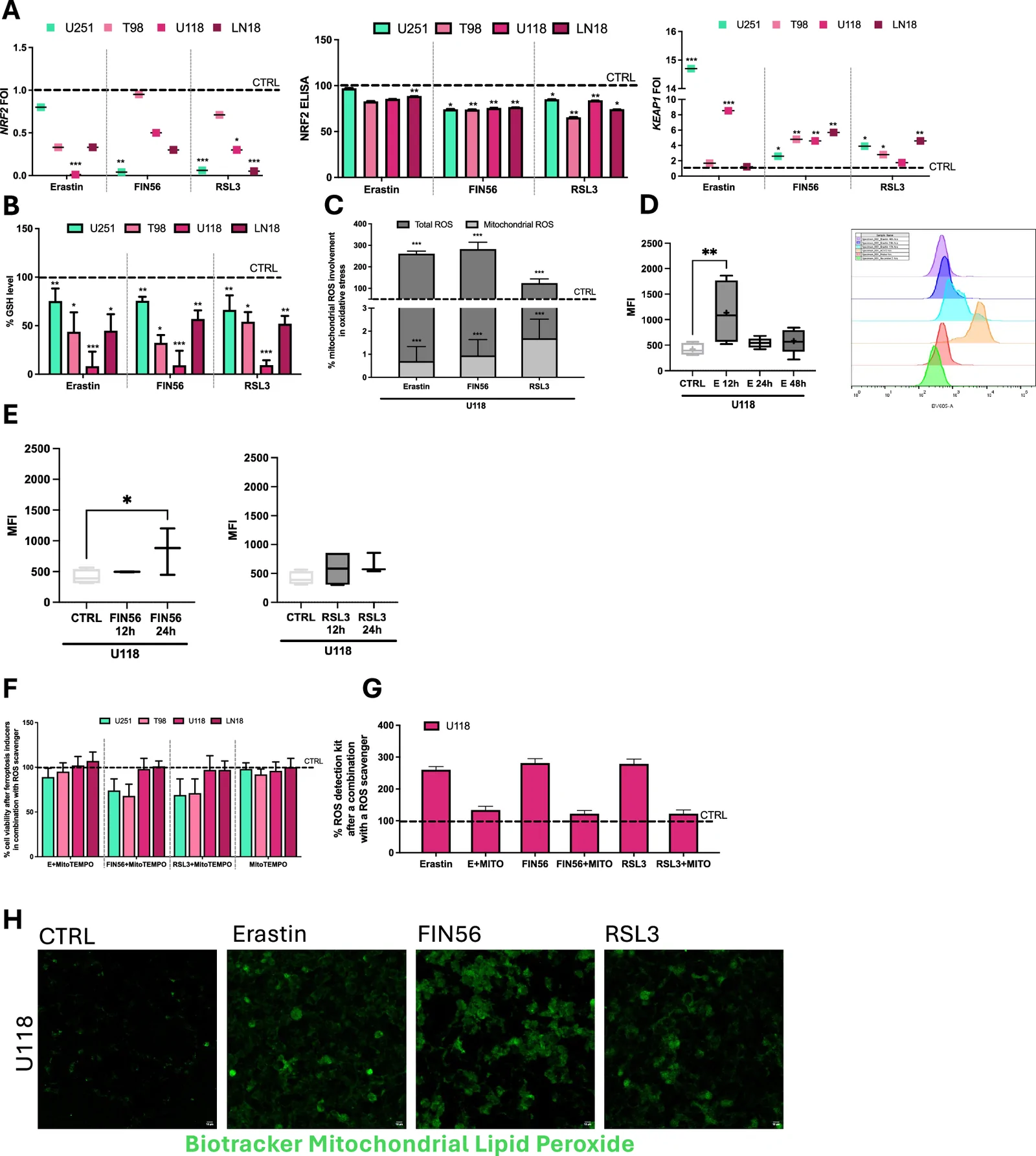
Redox impairment by ferroptosis inducers involves mitochondrial ROS and GSH depletion in glioblastoma. **A** mRNA expression of NRF2 and its negative regulator KEAP1, along with NRF2 protein levels (ELISA), following treatment with Erastin, FIN56, or RSL3. **B** Intracellular reduced glutathione (GSH) levels measured by colorimetric assay indicate antioxidant depletion. **C** Assessment of total versus mitochondrial ROS levels, showing selective mitochondrial ROS accumulation (MitoSOX). **D**, **E** Time-course analysis of mitochondrial ROS production after treatment. Mitochondrial ROS were measured by flow cytometry at 12, 24, and 48 h (Erastin) or 12 and 24 h (FIN56, RSL3). Boxplots show mean fluorescence intensity (MFI); representative histograms are shown in the right panel. **F**, **G** Cell viability and total ROS levels after combined treatment with ferroptosis inducers and the mitochondrial ROS scavenger MitoTempo, showing partial rescue and confirming mitochondrial involvement. **H** Confocal microscopy using a mitochondrial lipid peroxidation probe shows increased signal upon treatment; representative images and fluorescence quantification are reported. Scale bars are indicated in the panel. Data are presented as mean ± SD from at least three independent biological experiments (*n* = 3), each performed in technical triplicates unless otherwise specified. Statistical analyses were performed using two-way ANOVA followed by appropriate post hoc tests for multiple comparisons, or unpaired two-tailed Student’s *t* test for pairwise comparisons, using GraphPad Prism 11. A *p* value < 0.05 was considered statistically significant.

Focusing on the involvement of oxidative stress, NRF2 was analyzed and found to be impaired at both the molecular and protein levels by all three ferroptosis inducers with a concurrent upregulation of *KEAP1* that interacts with *NRF2* inhibiting its activity (Fig. 3A). In parallel, further confirming redox imbalance, Glutathione (GSH) levels were also affected by the entire panel of drugs used (Fig. 3B). This correlated with a more pronounced oxidative stress, both globally and at the mitochondrial level (Fig. 3C). To explore the individual contribution of ROS originating from different cellular compartments, we initially analyzed mitochondrial ROS using the MitoSOX probe through FACS; in particular, the time-dependent mitochondrial ROS release was further investigated in the U118 cell line as representative resistant to TMZ (as shown for other cell lines in Supplementary 4). More in detail, the Erastin drug induces a peak in mitochondrial ROS release at 12 h post-treatment, despite exerting its final biological effect on cell viability at 48 h (Fig. 3D). In contrast, the other two drugs reach their maximum ROS release at 24 h, coinciding with their biological effect, likely due to the different pharmacological targets involved (Fig. 3E). To better define the relationship between ferroptosis activation and oxidative stress, treatment with the ROS scavenger MitoTEMPO was included. Accordingly, ROS analysis demonstrated a significant reduction in ROS levels after treatment with the scavenger (Fig. 3F). Cell viability assays revealed that ROS scavenging significantly impaired the efficacy of the treatments, indicating that ROS generation is required for ferroptosis execution (Fig. 3G). Using confocal imaging, mitochondrial lipid peroxidation was further confirmed by fluorescent probe analysis (Fig. 3H). These results highlight the central role of oxidative stress, particularly mitochondrial ROS, in mediating ferroptosis. Targeting ROS dynamics may represent a critical leverage point in modulating ferroptosis-based therapies. The impairment of NRF2 signaling by ferroptosis inducers, along with the rescue effect observed with ROS scavenging, underscores the functional dependency of ferroptotic cell death on redox imbalance.

### Multi-omics integration highlights metabolic remodeling

**Fig. 4:**
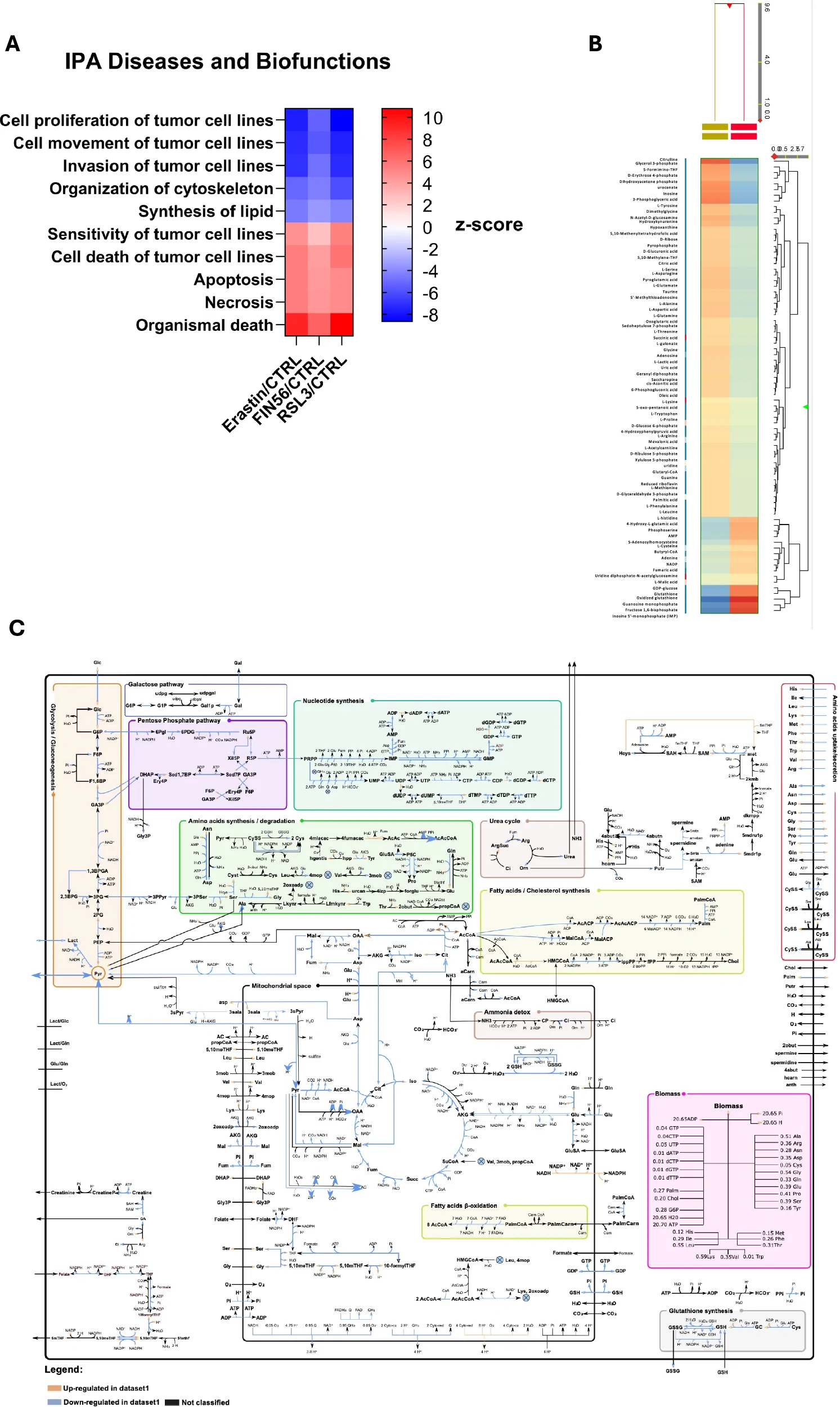
Integrated proteomic and metabolomic analyses reveal distinct alterations induced by ferroptosis inducers. **A** Heatmap of diseases and biological functions showing significantly enriched terms (*p* < 0.05) in datasets comparing Erastin, FIN56, and RSL3 treatments versus control (CTRL). Red and blue indicate predicted activation and inhibition, respectively, based on z-score values (activation: z ≥ 2; inhibition: z ≤ −2). **B** Hierarchical clustering heatmap showing significantly altered intracellular metabolites in U118 cells treated with Erastin *(A)*, FIN56 *(B)*, and RSL3 *(C)*, as detected by LC-MS. Metabolites were selected based on unpaired *t* test analysis with FDR correction (*p* value < 0.05). Color scale indicates log2 transformed-relative abundance, increasing from blue (low) to red (high). **C** ENGRO2 map of RPS- and RAS-enriched reactions in the Erastin vs CTRL condition. Arrow bodies are colored according to RAS (orange, upregulation; blue, downregulation), while arrowheads reflect RPS using the same color scale. Reactions lacking sufficient data are shown in black. Statistical analyses for metabolomic data were performed using unpaired two-tailed Student’s *t* test with FDR correction. A *p* value < 0.05 was considered statistically significant.

For proteome analysis, liquid chromatography coupled with electrospray ionization tandem mass spectrometry (LC-ESI-MS/MS) with TMT isobaric labeling was employed. A total of 3219 proteins were identified across all comparisons. Of these, 411 proteins in Erastin-treated cells, 235 in FIN56-treated cells, and 510 in RSL3-treated cells were differentially expressed compared to the control (CTRL), as determined by ANOVA followed by Tukey’s test with FDR correction (*p* value < 0.05). The identification data for the differentially expressed proteins (DEPs) detected by LC–ESI–MS/MS are provided in the Supplementary Files, available in the UNIMI Dataverse repository. Ingenuity Pathway Analysis (IPA) of the “Diseases and Bio Functions” categories significantly enriched among DEPs in all three treatments compared to the control revealed a shared pattern of biological response. Specifically, based on the expression pattern of changed proteins, the following biofunctions were predicted to be inhibited: *cell proliferation of tumor cell lines, cell movement of tumor cell lines, invasion of tumor cell lines, organization of cytoskeleton, and lipid synthesis*. In contrast, the following biofunctions were strongly predicted to be activated: *sensitivity of tumor cell lines, cell death of tumor cell lines, apoptosis, necrosis*, and *organismal death*. The heatmap can be consulted in Fig. 4A.

Semi-targeted metabolomics analysis revealed distinct overall metabolic profiles across the different drug treatments. Hierarchical clustering in Fig. 4B and Supplementary 5 illustrates the metabolites that are differentially abundant between the control and each treatment condition, as determined by unpaired t-tests with FDR correction (*p* value < 0.05). We identified 80, 35, and 53 significantly differentially abundant metabolites in cells treated with Erastin, FIN56, and RSL3, respectively, compared to control conditions. Among these, Erastin treatment induced the most pronounced metabolic alterations (Fig. 4B). Specifically, it led to a general accumulation of various metabolites, including several amino acids, multiple intermediates of the pentose phosphate pathway and metabolites from the first branch of the TCA cycle. Conversely, only a limited number of metabolites showed decreased abundance, primarily those involved in glutathione metabolism and intermediates of the second branch of the TCA cycle. On the other hand, FIN56 and RSL3 treatments induced relatively mild overall metabolic effects. In FIN56-treated cells, significant changes were predominantly confined to glutathione metabolism, as well as glycolysis (Supplementary 5). RSL3 similarly impacted these pathways (Supplementary 5), but exerted a broader and more pronounced metabolic shift. Notably, among the metabolites with statistically significant differences were several components associated with the Warburg Effect Superpathway.

To gain a more comprehensive and integrated understanding of the metabolic effects of the drugs, we used an extension of Marea4Galaxy [18] to map proteomics and metabolomics on the ENGRO2 metabolic network [19]. The output map comparing Erastin vs CTRL is illustrated in Fig. 4C. Taken together, the integrated analysis of proteomic and metabolomic data shows a general downregulation of central metabolic pathways at the protein level. Notably, proteomic data revealed a significant downregulation of mitochondrial proteins, including components of the TCA cycle, oxidative phosphorylation, and amino acid metabolism. In contrast, the metabolomic profile appears less affected or slightly upregulated. This pattern suggests a global metabolic suppression at the enzymatic level, possibly reflecting a reduced metabolic activity. In this scenario, a decrease in key enzymes could lead to the accumulation of metabolites intermediates. The only notable exception is glutathione synthesis, which is consistently downregulated at both the proteomic and metabolomic levels. This consistent downregulation could reflect a true metabolic suppression of the pathway, indicating potential cellular vulnerability to oxidative stress.

### Ferroptosis-inducing drugs suppress tumor progression in an orthotopic glioma model

**Fig. 5:**
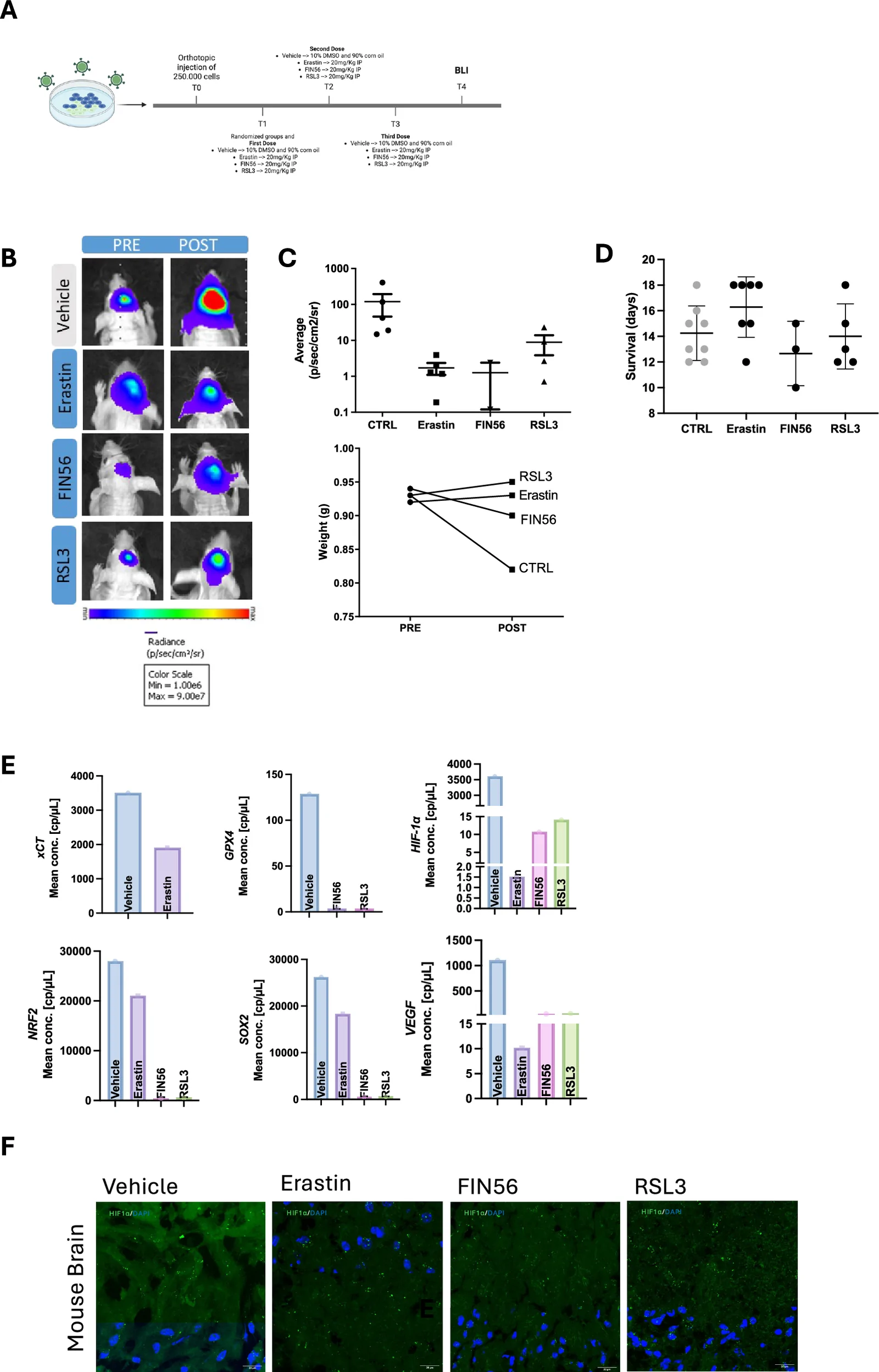
In vivo validation of ferroptosis induction and molecular effects in glioblastoma xenograft models. **A** Schematic representation of the in vivo experimental workflow. Luciferase-expressing U118 were implanted orthotopically in mice to enable bioluminescence-based tumor monitoring. Treatment schedule, dosing, and imaging time points are indictaed. **B** Representative bioluminescence images of mice bearing tumors, captured before (PRE) and after (POST) treatment with vehicle, Erastin, FIN56, or RSL3. Bioluminescence signal indicates tumor viability and metabolic activity. **C** Tumor burden was quantified by spectrophotometric analysis of brain tissues. ROI-based analysis of in vivo imaging revealed reduced tumor signal intensity in treated groups. Body weight was monitored before and after treatment to assess systemic toxicity. **D** Survival rate analyzed in treated-groups vs untreated was expressed in days. **E** Ex vivo analysis of brain tumor tissues by digital droplet PCR revealed altered expression of ferroptosis-related genes, angiogenesis- and hypoxia-related genes (e.g., *GPX4*, *SLC7A11*, *NRF2*) following treatment. **F** Representative confocal immunofluorescence images of brain sections showing expression and localization of HIF1α proteins in control and treated tumors. Data are expressed as mean ± SD. A minimum of *n* = [8] animals was used. Statistical analyses were performed using GraphPad Prism 11. Antibodies, fluorescence channels, and scale bars are detailed in figure panels.

To translate the experimental findings from bench to in vivo experimentation, an orthotopic glioma model using the U118-luc cell line was set up (Fig. 5A). All three drugs were shown to slow down the tumor growth (Fig. 5B). In fact, luciferase signal increase was higher in control group respect to treated ones. Consistently, ROI-based quantification demonstrated a reduction in bioluminescent signal intensity in mice treated with ferroptosis inducers respect to controls (Fig. 5C). Even if these differences did not reach statistical significance due to the small dimension of groups (*p value* = 0.06 for Erastin, *p value* = 0,22 for RSL3, *p value* = 0.38 for FIN56), these findings were promising, especially for Erastin treatment. Another noteworthy observation concerns disease progression: pharmacologically treated mice had a trend to exhibit less weight loss or even maintain the weight, consistent with a better overall health condition (Fig. 5C). As shown in Fig. 5D, survival data corroborate the previously reported findings, indicating a promising effect of Erastin, albeit with only a modest difference compared to the control group. At the endpoint, molecular analyses were conducted on harvested brain tissues to assess key markers. The expression levels of key pharmacological targets were first quantified. *xCT* was significantly reduced in mice treated with Erastin, and *GPX4* was decreased in those treated with RSL3 and FIN56, compared to the vehicle group. These data validated the expected effect of the three drugs, showing that they were able to reach the brain and exert their function. *HIF-1α* levels were also lower across all treated groups, along with a reduction in *VEGF*, *SOX2*, and *NRF2* expression (Fig. 5E). Finally, confocal microscopy analysis confirmed the reduction of the expression and the nuclear localization of HIF-1α protein in ex vivo brain slices of treated mice (Fig. 5F). Altogether, these findings confirm the therapeutic potential of ferroptosis inducers in glioma, not only by impairing tumor progression in vivo, but also by targeting their key molecular pathways.

## Discussion

Resistance remains one of the main obstacles in the treatment of GBM. The failure of therapeutic strategies is the result of a complex interplay of molecular and biochemical factors. In this context, several studies suggest that the increasing oxidative stress may present a novel therapeutic approach, even in GBM. There is growing interest in the role of oxidative stress in cancer treatment, particularly in leveraging it to induce cytotoxicity in cancer cells [9]. Recent studies discuss the mechanisms through which oxidative stress is induced in cancer cells and how it contributes to cytotoxic effects, either through direct ROS generation, ferroptosis, or activation of death pathways. Therapeutic strategies aimed at enhancing oxidative stress in tumors are emerging as promising approaches in oncology, often complementing existing therapies to overcome resistance and improve treatment efficacy [8, 20]. The present study offers compelling evidence that ferroptosis represents a vulnerable axis in TMZ-resistant glioma and that its pharmacological induction could represent a promising therapeutic option. By characterizing the molecular landscape of TMZ-responsive versus TMZ-resistant glioma cell lines, we identified HIF-1α overexpression as a hallmark feature [20, 21]. This hypoxia-inducible transcription factor is widely known to orchestrate adaptive responses to hypoxic stress and is here implicated in modulating ferroptosis resistance mechanisms. Importantly, TMZ-resistant cells exhibited lower levels of lipid peroxidation and higher antioxidant capacity, mediated through elevated GSH and NRF2 modulation, indicating an active ferroptosis-resistant phenotype. In line with this, the silencing of *GPX4* and *xCT*, two core ferroptosis regulators, resulted in a marked reduction in cell viability. Notably, while HIF-1α levels correlate with GPX4 and xCT expression, our data suggest that this regulation occurs largely through an indirect mechanism, in which the key mediator is the cellular ROS level and its modulation by pharmacological treatments, rather than direct transcriptional control. These findings not only validate the central role of these targets in ferroptosis escape but also highlight their translational potential as therapeutic targets. Recent studies have demonstrated that pro-oxidant treatments, such as those increasing ROS levels, can induce cytotoxicity in tumor cells, highlighting both the mechanisms by which oxidative stress causes cell death and the challenges in translating these strategies into effective treatments [17]. Other evidence provides an overview of how increasing ROS in cancer cells can lead to cytotoxic effects and discusses strategies for inducing oxidative stress to promote cancer cell death, including through drugs that target antioxidant defenses. In GBM, therapy-resistant cells exploit NRF2-mediated antioxidant responses, including upregulation of GPX4 and xCT, to maintain redox balance and evade ferroptotic death [11]. HIF‑1α further modulates redox metabolism, likely through indirect effects on ROS and metabolic remodeling, rather than direct transcriptional control of *GPX4*/*xCT*. Collectively, these findings indicate that oxidative stress can be modulated to enhance cancer cell cytotoxicity, particularly using pro-oxidant drugs that increase ROS production to levels that exceed the cancer cell’s antioxidant capacity, representing a potential strategy to treat cancer [22].

In this paper, we examined the induction of ferroptosis as a potential new therapeutic strategy in glioma. Therefore, this study proposes Erastin, FIN56, and RSL3 as valid alternatives to conventional chemotherapy in GBM. Mechanistically, all three ferroptosis-inducing drugs resulted in the downregulation of *NRF2*, *GPX4*, *ATF4*, and *FSP1*, while upregulating *KEAP1*, the *NRF2* inhibitor, thereby tilting the cellular redox balance toward ferroptosis [23–25]. Furthermore, these agents consistently reduced HIF-1α expression and activity [11]. The pivotal role of HIF-1α was functionally validated using treatment with DFX, a hypoxia mimetic that stabilizes nuclear HIF-1α, effectively reversed the ferroptosis-induced molecular signatures and therapeutic effects, confirming that HIF-1α suppression is necessary for ferroptosis activation in this context [11, 26]. By integrating metabolomic and proteomic analyses, we demonstrated that all tested ferroptosis inducers ultimately converge on disrupting core metabolic processes in glioblastoma cells. The metabolomic profiles suggest that the compounds share common targets but also display drug-specific metabolic signatures. Erastin induces extensive metabolic remodeling, characterized by amino acid accumulation, activation of the pentose phosphate pathway, and increased early TCA cycle activity, indicative of oxidative stress and anabolic compensation. The marked depletion of glutathione further supports ferroptosis-driven redox collapse. In contrast, FIN56 and RSL3 produced more modest metabolic alterations. FIN56 primarily impacted glutathione metabolism and glycolysis, while RSL3 elicited broader changes, affecting redox balance and multiple metabolites associated with the Warburg Effect, suggesting mitochondrial dysfunction and altered energy metabolism [27]. When integrated with proteomic data, these metabolic alterations point to a shared disruption of mitochondrial function and respiratory chain imbalance. This mitochondrial stress contributes to oxidative damage and activation of ferroptotic cell death pathways, even in chemoresistant glioblastoma models [28]. The creation of a chemoresistant and engineered orthotopic GBM model allowed us to monitor the effect of the three inducers administered systemically over time. Treatment with ferroptosis inducers reduced tumor burden and bioluminescence intensity but also showed the trend toward improving systemic health markers, such as body weight maintenance; moreover, for Erastin group, the treatment showed a propensity in delaying the achievement of the humane endpoint (that is the time to stop the experimentation to reduce the animal pain). These data therefore suggest that our engineered and orthotopic implanted model is valid for testing new therapeutic approaches, and that the three drugs seems to be effective in slowing down tumor growth in our GBM model. Even if the small dimensions of the groups, the in vivo results are very encouraging, especially when combined with the ex vivo characterization of tumor masses. In fact, the ex vivo analyses clearly demonstrated the specific activity of each drug in modulating their well-defined target, demonstrating that they reach the brain where they exert their certain effect. Moreover, they also clarify that the antiproliferative effect of the three drugs, in terms of the molecular setup of ferroptosis and the HIF-1 biomarker, is consistent [16]. Ex vivo studies showed that the expression of the ferroptosis-associated gene panel aligned with pathway activation. In the tumor, the expression of HIF-1 was also downregulated, further confirming its role as a biomarker in vivo.

## Conclusions

Our findings indicate that resistance to ferroptosis in glioma is closely associated with HIF-1α signaling and redox homeostasis, and that simultaneous disruption of these pathways may restore sensitivity to ferroptosis. This study provides a mechanistic basis for integrating ferroptosis inducers into glioma treatment regimens, especially in refractory or hypoxia-adapted tumors. Future research should aim to translate these findings into clinical application and explore combination therapies that further compromise ferroptosis-resistance mechanisms in GBM.

## Materials and methods

### Cell lines and reagents

U251 TMZ-responsive human glioma cell line was provided by ICLC (Ospedale San Martino, Genoa, Italy) in 2023 instead T98, U118 and LN18, TMZ-resistant human glioma cell lines, were purchased in 2023 from ATCC (American Type Culture Collection). U251 and T98 were maintained in RPMI supplemented with 10% heat-inactivated fetal bovine serum, 1% penicillin and streptomycin (50 IU/ml), 2 mM glutamine (all Euroclone, Milan, Italy) in a humidified atmosphere of 5% of CO_2_ at 37 °C. U118 and LN18 were maintained in DMEM high glucose supplemented with 10% heat-inactivated fetal bovine serum, 1% penicillin and streptomycin (50 IU/ml), 2 mM glutamine (all Euroclone, Milan, Italy) in a humidified atmosphere of 5% of CO_2_ at 37 °C. ^In^ Table 1 it is shown the characterization of the four cell lines used in all the experiments performed.

**Table 1:** Comprehensive characterization of the glioblastoma (GBM) cell lines employed in the study.

Cell line	Classification	PTEN	P53	IDH1	Methylation % MGMT
U251	GBM	Mut	Mut	Wt	65%
T98	GBM	Mut	Mut	Wt	40%
U118	GBM	Mut	Mut	Wt	Hemi-methylated
LN18	GBM	Wt	Mut	Wt	25%

This table includes key information on the cell line origin, genetic profile, treatment responsiveness, and specific markers analyzed during the experiments.

### Cell viability

For viability tests, were plated 50,000 cells in a 24-well, and were added 500 μl of complete medium as final volume. For RNA extraction, 500,000 cells were plate in a 6-wells in a final volume of 2 ml. In vitro treatments were performed as reported in Table 2. After the treatment, the cells were counted using a Burker’s Chamber with Trypan Blue exclusion test and data were expressed as percentage viable cells.

**Table 2:** Overview of the treatments applied to glioblastoma (GBM) cell lines in the experiments.

Treatment	Stock	Final concentration	Time
Temozolomide (TMZ)	10 mM	100 μM	24 h
Erastin (E)	9,14 μM	2 μM	48 h
FIN56	10 mM	1 μM	24 h
RSL3	10 mM	100 nM	24 h
Deferoxamine (DFX)	76 mM	100 μM	24 h
Mito-TEMPO	19,6 mM	25 μM	24 h

This table includes the specific drugs, concentrations, treatment durations, and relevant experimental conditions used to assess the response of both sensitive and resistant GBM cell lines.

### Lipid Peroxidation

The cells were plated in a 100 mm petri dis. Cells were maintained in complete medium, or with the addiction of treatment. Lipid Peroxidation (MDA) Assay Kit (Colorimetric/Fluorometric) (Cat. No. ab118970, abcam, Italy) was performed on control cells and on treated cells. In the lipid peroxidation assay, the malondialdehyde (MDA) in the sample reacts with thiobarbituric acid (TBA) to generate MDA-TBA adduct. The MDA-TBA adduct can be easily quantified fluorometrically (Ex/Em=532/553 nm). The results were analysed by comparing the treated samples with the control.

### RNA extraction and real-time PCR

RNA was extracted using Trizol (Zymo Research) protocol. Total RNA was reverse transcribed to cDNA using a commercial kit from Takara (PrimeScript RT Master Mix). The real-time PCRs were performed in triplicate for each data point by using the Sybr Green (2X Green Fast qPCR Mix, Fisher molecular biology FMB) technique; the oligonucleotides (Eurofins, Milan, Italy) used are shown in Table 3. Target mRNA content changes in relation to the β-ACTIN (*β-ACT)* housekeeping gene were determined using the ΔΔCt Method (and represented as FOI, fold of induction, compared to control levels) [29].

**Table 3:** Primer sequences for the genes analyzed in the experiments.

Gene	Primer sequence Fw	Primer sequence Rw
*β-ACT*	CACCATTGGCAATGAGCGGTTC	AGGTCTTTGCGGATGTCCACG
*HIF-1α*	TGATTGCATCTCCATCTCCTACC	GACTCAAAGCGACAGATAACACG
*FSP1*	GGAGACAGGGTTCGCCAAAA	CCCGTGGCCAGGATAAGATG
*GPX4*	CGCAACGATGTTGCCTGGAACTTT	AGGCTCGATGTCAATGGTCTGGAA
*xCT (SLC7A11)*	ATGCAGTGGCAGTGACCTTT	GGCAACAAAGATCGGAACTG
*NRF2*	CAGCGACGGAAAGAGTATGA	TGGGCAACCTGGGAGTAG
*KEAP1*	TTCTGGGGATCCATG	CTCCAAGGACGTAGATTCTC
*ATF4*	TTCTCCAGCGACAAGGCTAAGG	CTCCAACATCCAATCTGTCCC

This table lists the specific sequences of forward and reverse primers used for PCR amplification of genes relevant to glioblastoma pathophysiology, including those involved in treatment response and oxidative stress pathways.

### Digital PCR

Digital PCR reactions consisted of 11 µL reaction mixture per well containing QIAcuity 4× Probe PCR master mix (Qiagen), primers (Fw and Rw), water for molecular biology (ITW Reagents), and 15 ng RNA template. Assembled reactions were transferred into QIAcuity 8.5k 24-well Nanoplates (Qiagen) for partitioning using the Qiagen Standard Priming Profile. The QIAcuity Software Suite (Qiagen, version 2.1.7) was used to determine sample thresholds using positive, negative, and no-template control wells (NTCs) with the manual global threshold approach that is based on the amplitude signal observed in negative control samples.

### Transfection

The transfection was undertaken culturing 50.000 cells in each well in a 24-well plate for cell viability and 500,000 cells in a 6-well plate for RNA extraction. Glioma cells were transfected with 10 nM of xCT or 10 nM of GPX4 siRNA by Qiagen (Milan, Italy), or a scrambled negative control (Product No. 1027280, Qiagen) using the Attractene Transfection Reagent (Cat. No. 301005, Qiagen) as indicated by the manufacturer for 24 h.

### GSH detection assay

Detection and quantification of Glutathione (GSH) was performed by the commercially available GSH-Glo™ Glutathione Assay (Promega). Data were expressed as Relative luminescence unit (RLU) in treated cells compared to untreated cells, according to the manufacturer’s instructions.

### Mitochondrial ROS assay

Mitochondrial ROS content was quantified by using MitoSOX™ Mitochondrial Superoxide Indicators kit (Cat. No. M36005, Invitrogen, Thermo Fisher Scientific, Milan, Italy). 500.000 cell were plated in each well in a 6-well plate, and the treated cells were stained with a probe following the manufacturer’s instructions. The experimental conditions included: untreated cells and untreated stained cells as negative controls, H2O2 stimulated samples as positive control, and drugs treated cells. Data acquisition was performed on a blue- and violet-laser–equipped BD FACSCelesta flow cytometer, and flow cytometry data were analyzed using FlowJo v11 software. Cellular events were initially gated based on forward and side scatter (FSC/SSC) properties to exclude debris and events with abnormal scatter characteristics. Doublets were excluded by sequential gating on forward scatter area versus height (FSC-A/FSC-H). Fluorescence intensity data were displayed using biexponential scaling, and mitochondrial ROS levels were quantified as the mean fluorescence intensity (MFI) in the BV605-A channel.

### ELISA—enzyme-linked immunosorbent assays

An ELISA-based kit (TransAM Kit, Vinci-Biochem, Vinci, Italy) was used to detect and quantify HIF-1α transcriptional factor activity and NRF2 transcription factor in accordance with the manufacturer’s instructions. The data are expressed as the amount of HIF-1α protein in nuclear or cytoplasmatic extract (OD 450 nm). The concentration of xCT in glioma cell-conditioned medium was quantified using an ELISA kit (Human SLC7A11 (Cystine/glutamate transporter), CABRU sas, Monza, Italy) in accordance with the manufacturer’s protocol. The data are expressed in pg/mL. The concentration of GPX4 in glioma cell-conditioned medium was quantified using an ELISA kit (GPX4 Fluorogenic Assay Kit, BPS Bioscience, San Diego, USA) in accordance with the manufacturer’s protocol. The data are expressed in percentage (%) of activity.

### Biotrackers

In total, 60,000 cells were plated in a 12-well in adherence with RPMI or DMEM supplemented with 10% heat-inactivated fetal bovine serum, 1% penicillin and streptomycin (50 IU/ml), 2 mM glutamine (all Euroclone, Milan, Italy) in a humidified atmosphere of 5% of CO_2_ at 37 °C. After 24 h, Erastin, FIN56 or RSL3 were added to the culture media at the concentration of 2 μM, 1 μM and 100 nM, respectively. Once treatment had expired, the wells were washed with PBS and incubated at 37 °C for 30 min. Then the living adherent cells were observed under a confocal microscope with a 10x objective (STELLARIS Confocal Microscope Platform Leica Microsystems). The fluorescence intensity was calculated by using the ImageJ software (NIH, New York, NY, USA).

### Immunofluorescence

A total of 60.000 GBM cells were seeded on round glass slides and cultured in adherence with RPMI or DMEM supplemented with 10% heat-inactivated fetal bovine serum, 1% penicillin and streptomycin (50 IU/ml), 2 mM glutamine (all Euroclone, Milan, Italy) in a humidified atmosphere of 5% of CO_2_ at 37 °C. After 24 h, Erastin, FIN56 or RSL3 were added to the culture media at the concentration of 2 μM, 1 μM and 100 nM, respectively. The previous treatments were administered either individually or in combination with DFX. After 24 h or 48 h, concerning to the treatment, the cells were washed by PBS and fixed for 30 min at 37 °C with 4% paraformaldehyde (PFA). After that, the round glass slides were washed with PBS and permeabilized with PBS/FBS 5%/Triton 0.3% solution for 10 min at 4 ^◦^C. After washing with PBS, the cells were incubated with a blocking buffer composed of BSA 2% in PBS tween 0.1% for 30 min at room temperature. The cells were incubated overnight at 4 ^◦^C with the HIF-1α (sc-13515, Santa Cruz Biotechnology, INC.) primary antibody. Thereafter, the cells were labeled as 488 Alexa Fluor-conjugated secondary antibodies (Invitrogen, Waltham, MA, USA) for 30 min at room temperature in the dark. The nuclei were blue counterstained with DAPI (D1306, Invitrogen). Next, the cells were washed with PBS, and the slides were closed with glass coverslips using a clear mount solution (Invitrogen). Images were acquired at 63× magnification, to which a 1.65x zoom was added, with a STELLARIS Confocal Microscope Platform, Leica Microsystems. Cell morphological analysis was performed by using the ImageJ software (NIH, New York, NY, USA).

### In vivo imaging study design

Animal experiments were carried out in compliance with the institutional guidelines for the care and use of experimental animals (European Directive 2010/63/EU). The protocol was approved by the Animal Use and Care Committee of the University of Milan and authorized by the Italian Ministry of Health. U118-luc human glioma cells express the luciferase reporter gene under a constitutive promoter (PGK). Before orthotopic injection, U118-Luc cells were tested for responsiveness to ferroptosis inducers. Mice were kept in appropriate cages in an environment of 23 ± 1 °C and 50 ± 5% humidity, with a 12 h light/dark cycle, and were fed ad libitum with a standard diet (Rat/Mouse Maintenance V1534 diet, sniff Spezialdiäten, Soest, Germany) for one week to allow for acclimatization. Subsequently, they were anesthetized with a mixture of ketamine (100 mg/kg, Lobotor, Acme, Cavriago, Italy) and xylazine (8 mg/kg, Rompun, Elanco, Sesto Fiorentino, Italy) intraperitoneally injected in PBS—and underwent surgery. The orthotopic murine model of glioma was established, as described by our group [16], by stereotaxic injection of 2.5 × 10⁵ U118-Luc cells in 2 μl of phosphate-buffered saline (PBS) into 7- to 8-week-old female nude mice (Harlan Laboratories, USA) on day 0. Following surgery, mice were monitored for recovery until complete awakening. For the detection of bioluminescence, mice were anesthetized with the ketamine/xylazine mixture. Then, 150 mg/kg of luciferin (Beetle Luciferin Potassium Salt, Promega, substrate of the luciferase enzyme) was intraperitoneally injected into tumor-bearing mice for bioluminescence imaging (BLI). After biodistribution, bioluminescence was acquired by placing the mice in the light-tight chamber of the IVIS SPECTRUM/CT instrument (PerkinElmer Life Sciences). Images were scaled and analyzed, by applying the same region of interest (ROI), after completion of all acquisitions, using the Living Image Software 4.7.4 (PerkinElmer). The ROI positioning was done in a blinded way, using only the anonymous photography of the mice instead of bioluminescent signal, to avoid any bias due to the operator. Data were presented as average radiance (photons/second/square centimeter/steradian), which is a calibrated measurement of photon emission. After tumor establishment, mice were assigned to experimental groups using a stratified randomization approach based on tumor size to ensure comparable baseline tumor burden across the four groups (the mice whose tumor signal did not increased over time or whose signal was near the background level were excluded by following grouping), each receiving 3 intraperitoneal doses every other day of four different treatments: control (receiving vehicle: 10% DMSO, 90% corn oil, *n* = 8), Erastin (20 mg/kg, *n* = 7), FIN56 (20 mg/kg, *n* = 3), or RSL3 (20 mg/kg, *n* = 5). With this sample size, the power of the study was 0.8, with an effect size of 0.6 and an α error of 0.05.

### Metabolite extraction from cell culture for LC-MS

At the end of treatment, cells were washed with a 0.9% NaCl solution (Sigma-Aldrich, St. Louis, Missouri, USA) and subsequently quenched with an ice-cold mixture of 70:30 acetonitrile:water (both from Honeywell, Wabash, Indiana, USA). The plates were then placed at −80 °C for 10 min, followed by collection through scraping and sonication (twice for 5 s each, at 70% power). After centrifugation at 12,000 x *g* for 10 min, the supernatant aqueous phases were collected in a glass insert and dried using a centrifugal vacuum concentrator (Concentrator plus/Vacufuge plus, Eppendorf, Hamburg, Germany) at 30 °C for approximately 2.5 h. Finally, the samples were resuspended in 150 μL of H2O before analysis.

### LC-MS metabolic profiling

Liquid chromatography (LC) separation was conducted using an Agilent 1290 Infinity UHPLC system with an InfinityLab Poroshell 120 PFP column (2.1 × 100 mm, 2.7 μm; Agilent Technologies, Santa Clara, CA, USA). The mobile phase A consisted of water with 0.1% formic acid (both from Honeywell, Wabash, Indiana, USA), while mobile phase B comprised acetonitrile with 0.1% formic acid. The injection volume was 10 μL, and the LC gradient conditions were as follows: 0 min: 100% A; 2 min: 100% A; 4 min: 99% A; 10 min: 98% A; 11 min: 70% A; 15 min: 70% A; 16 min: 100% A with a 2-min post-run period. The flow rate was 0.2 ml/min, and the column temperature was maintained at 35 °C. Mass spectrometry (MS) detection was performed using an Agilent 6550 iFunnel Q-TOF mass spectrometer equipped with a Dual JetStream source, operating in negative ionization mode. The specific MS parameters were as follows: gas temperature: 285 °C; gas flow: 14 l/min; nebulizer pressure: 45 psig; sheath gas temperature: 330 °C; sheath gas flow: 12 l/min; VCap: 3700 V; Fragmentor: 175 V; Skimmer: 65 V; Octopole RF: 750 V. Active reference mass correction was achieved through a second nebulizer using masses with m/z values of 112.9855 and 1033.9881. Data acquisition spanned the range of m/z 60–1050.

### Metabolomics data analysis

Data were processed using MassHunter Profinder (v10.0.2, Agilent Technologies) with the Batch Targeted Feature Extraction workflow and the Agile 2 algorithm for peak detection and alignment. Metabolites were identified by matching accurate mass and retention time against a custom Agilent PCDL database, and only compounds with an ID score above 75 were included in the analysis. Metabolite intensities were normalized to total protein content to account for sample variability. Statistical analysis was performed using Mass Profiler Professional (v15.1, Agilent Technologies). Data were log₂-transformed and Pareto scaled to reduce the impact of very high or low values, keeping in the analysis only those metabolites detected in 100% of samples in at least one group for each comparison. Significant differences between conditions were assessed using unpaired *t* tests with Benjamini–Hochberg FDR correction (*p* < 0.05), and results were visualized using hierarchical clustering.

### Protein extraction from cell cultures

Cells were washed twice in ice cold PBS and collected by centrifugation for 5 min at 200 × *g*. Cell pellets were resuspended in five cell-pellet volumes of 100 mM triethylammonium bicarbonate (TEAB), 1% SDS, and sonicated twice for 5 s each, at 70% power. Cell lysates were clarified by centrifugation at 16,000 × *g* for 10 min at 4 °C and the protein concentration of the supernatant determined using the BCA Protein Assay Kit (Pierce, Product No. 23227).

### Tandem mass Tag (TMT) LC-MS/MS proteomic studies

One hundred μg of protein per condition were labeled with the TMT Mass Tagging Kit (Thermo Fisher Scientific) following the manufacturer’s protocol. Proteins were reduced with 200 mM tris(2- carboxyethyl) phosphine (TCEP), alkylated with 375 mM iodoacetamide, and purified by acetone precipitation. Pellets were resuspended in 100 μl of 50 mM TEAB, digested overnight at 37 °C with trypsin, labeled with TMT reagents, and mixed. The mixture was dried (SpeedVac) and resuspended in 750 μl of 0.1% trifluoroacetic acid (TFA). For deeper proteome coverage, the sample was fractionated into 8 fractions using the high-pH reversed-phase peptide fractionation kit (Pierce, #84868). Each fraction was centrifuged, dried (SpeedVac), and resuspended in 30 μL of 0.1% formic acid (FA). Four μl per fraction were injected and analyzed in technical duplicate on a Dionex Ultimate 3000 nano-LC system (Thermo) coupled to an Orbitrap Fusion™ Tribrid™ Mass Spectrometer with a nano-ESI source. Peptides were pre-concentrated on a PepMap 100 C18 trap column (0.3 × 5 mm) and separated on an EASY-Spray ES802A C18 column (25 cm × 75 μm, 3 μm, 100 Å) using a 120- min LC gradient (flow rate: 0.300 μL/min, 35 °C) from 4% to 95% mobile phase B (0.1% FA in acetonitrile). MS analysis was performed in positive ion mode using data-dependent SPS-MS3. Peptides were ionized at 1600 kV. Orbitrap MS1 scans were acquired at 120,000 resolution (375–1500 m/z; AGC 4 × 10⁵; max IT 50 ms). MS2 was triggered for precursors (z > 1) with dynamic exclusion (60 s, ±10 ppm), using quadrupole isolation (0.7 m/z), CID fragmentation (35% CE), and ion trap detection (AGC 1×10⁴, max IT 50 ms). SPS-MS3 scans (range 400–1200 m/z, 10 precursors) used a 2 m/z isolation window, HCD fragmentation (65% CE), Orbitrap detection (50,000 resolution; 100–500 m/z; AGC 5×10⁴; max IT 54 ms).

### Proteomics statistical data analysis

The raw files obtained from the analysis were processed using MaxQuant software (version 2.7.0.0, Max Planck Institute of Biochemistry, Germany) for protein identification and quantification. The resulting “protein groups” were analyzed using Perseus software (version 2.0.11, Max Planck Institute of Biochemistry) to determine statistically significant proteins. Statistical analysis was conducted by applying one-way ANOVA (*p* value < 0.05), followed by Tukey’s post hoc test, corrected for the Benjamini and Hochberg FDR to identify proteins that were significantly altered across the comparisons.

### Ingenuity pathway analysis

Functional and network analyses of statistically significant protein expression changes were performed through Ingenuity Pathway Analysis (IPA) software (Qiagen, Hilden, Germany, Summer Release 2024). The “core analysis” function was used for data interpretation through the analysis of diseases and biofunctions enriched with differentially regulated proteins. Then the “comparison analysis” function was used to visualize and identify significant functions across experimental conditions. *p* values were calculated using a right-tailed Fisher’s exact test. The activation z-score was used to predict the activation/inhibition of a disease and biofunctions. A Fisher’s exact test *p* value < 0.05 and a z-score ≤−2 and ≥2 were considered statistically significant.

### Mapping proteomics and metabolomics on the metabolic network

To project proteomics on metabolic level, we used the Reaction Activity Scores [30]. These features are originally used for mapping the gene expression data to a set of metabolic reactions, by means of the Gene Protein Reaction (GPR) rules. The GPR rules represent logical formulas describing how gene products concur to catalyze a given reaction, using Boolean operators. The AND operator is used when distinct genes encode different subunits of the same enzyme, whereas the OR operator is used for genes encoding isoforms of the same enzyme. The RAS scores are calculated by substituting the proteomics intensities in the corresponding GPRs. Then, the logical expressions are solved by taking the minimum level value when multiple genes are joined by an AND operator, and the sum of their values when multiple genes are joined by an OR operator. For this comparison, we uploaded the proteomics file (3158 proteins for 8 samples) on Marea4Galaxy and computed the RAS scores using the RAS generator module obtaining a total of 238 RASs, that is, reactions for which the RASA is available. In a similar way, to integrate metabolomics data, we used specific features called Reaction Propensity Scores (RPS). These scores are computed as follows:

$${RPS}=\log \left(\prod _{i{\rm{\epsilon }}S}{\left(\frac{\left[{S}_{i}\right]+\epsilon }{{n}_{i}}\right)}^{{v}_{i}}\right)$$

where S_i is the concentration of substrate i, ν_i is the stoichiometric coefficient of substrate i, n_i is the number of reactions in which the substrate i is involved, ϵ is a small quantity to avoid log 0. For this computation, we uploaded the metabolomics file (118 metabolites for 9 biological samples), and computed the RPS scores, obstaining a total of 131 RPSs.

We combined RAS and RPS with a scaffold core model of metabolism, called ENGRO2 [19], and integrated into a bioinformatic pipeline that allowed to generate a description of the metabolism with the aim of showing possible differences between two biological conditions. ENGRO2 contains 469 reactions, 395 metabolites, and 498 genes. For this model, 351 model reactions are associated with a gene-protein-reaction (GPR) rule. Moreover, for this model, a manually curated map is available for mapping statistically significant differences between the RAS and RPS of two groups of samples.

This tool, compared to the original version, offers the possibility to statistically compare both the RAS and RPS of two user-defined groups of samples across all metabolic reactions, and to visualize the identified differences on the metabolic map. RAS differences are mapped onto the arrow body, while RPS differences are mapped onto the arrowhead. Importantly, RPS data are directional: reversible reactions are treated as two distinct directional components, with the direction encoded in their reaction IDs.

### Statistical analysis

In vitro experiments were independently repeated at least three times (*n* = 3) and yielded reproducible results. Data are presented as mean ± SD from independent biological replicates, each performed in technical triplicates unless otherwise specified. Sample size was determined based on previous literature, laboratory experience, and preliminary experiments to ensure adequate sensitivity to detect biologically relevant differences.

Statistical analyses were performed using GraphPad Prism 11 (GraphPad Software Inc., San Diego, CA, USA). Statistical tests were selected based on the experimental design and data structure and were considered appropriate for each analysis. Data were assumed to follow a normal distribution, and parametric tests were applied accordingly. Variability within each group is reported as mean ± SD, and variance was comparable between groups being statistically compared.

Comparisons between two groups were performed using an unpaired two-tailed Student’s *t* test, while multiple group comparisons were analyzed using one-way or two-way analysis of variance (ANOVA), followed by Dunnett’s or Bonferroni’s post hoc tests, as appropriate.

Details of statistical tests and sample size are provided in the figure legends.

## Supplementary information

Supplemetary material

## Data Availability

Proteomic data: https://doi.org/10.13130/RD_UNIMI/CNOJ.
