# Supplementary material for: Ferroptosis-induced oxidative stress in therapy-resistant glioblastoma
Source: Cell Death Discov. 2026 Jun 26;12:366. doi: 10.1038/s41420-026-03227-3 (PMC13572394; doi:10.1038/s41420-026-03227-3)
Supplement: Supplementary file 1 [file 41420_2026_3227_MOESM1_ESM.pdf]

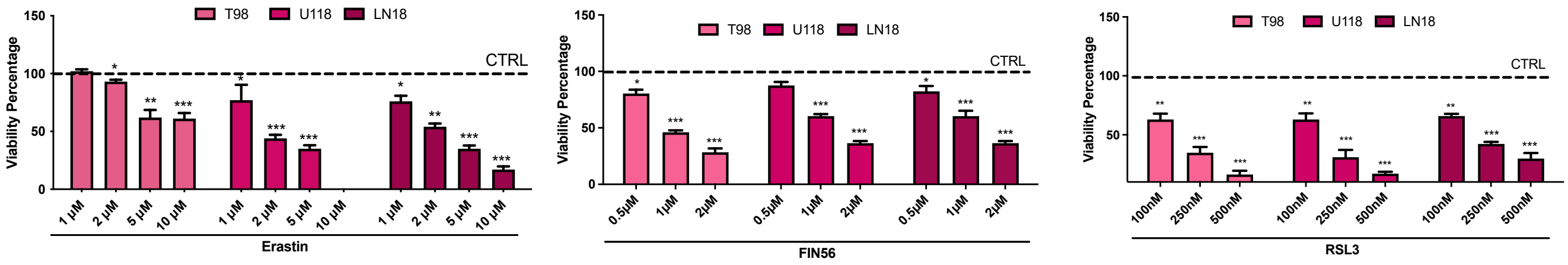

Supplementary 1. *Cell viability of drug-treated resistant glioblastoma multiforme (GBM) cells.*
Trypan blue exclusion assay was performed to assess cell viability upon increasing concentrations of the tested compounds (Erastin, FIN56 and RSL3) in therapy-resistant GBM cell lines (T98, U118, LN18). Data are presented as mean ± SD from three independent experiments. Statistical analysis was performed using one-way ANOVA with appropriate post-hoc tests.

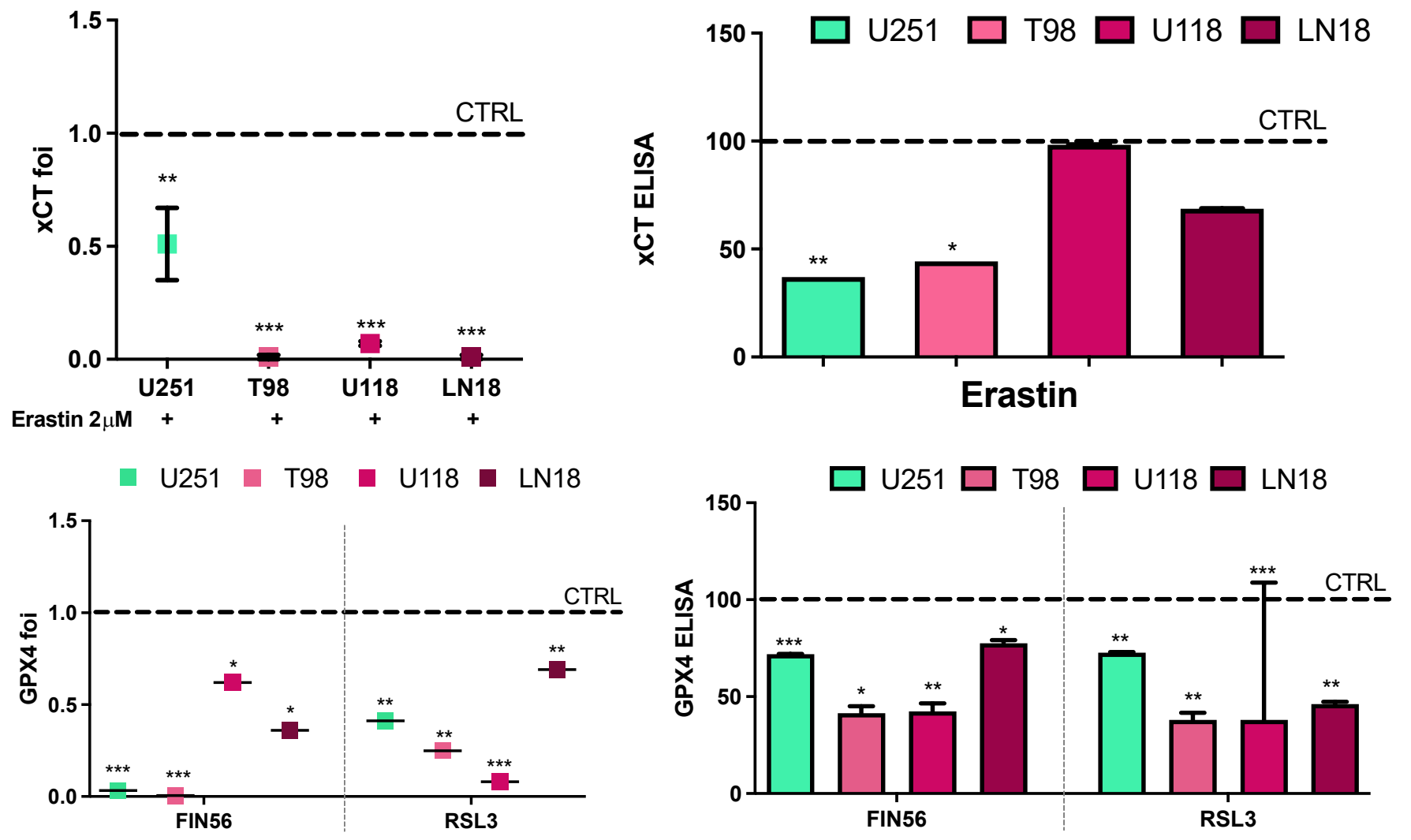

Supplementary 2. *Gene expression and protein quantification of drug targets in resistant GBM cells.*
Real Time PCR analysis of mRNA expression levels of the two pharmacological targets (xCT and GPX4) in GBM cells treated with the indicated compounds (Erastin, FIN56 and RSL3). Quantification of the corresponding protein levels by ELISA. Data represent mean ± SD of three biological replicates. *P* values were determined by unpaired t-test.

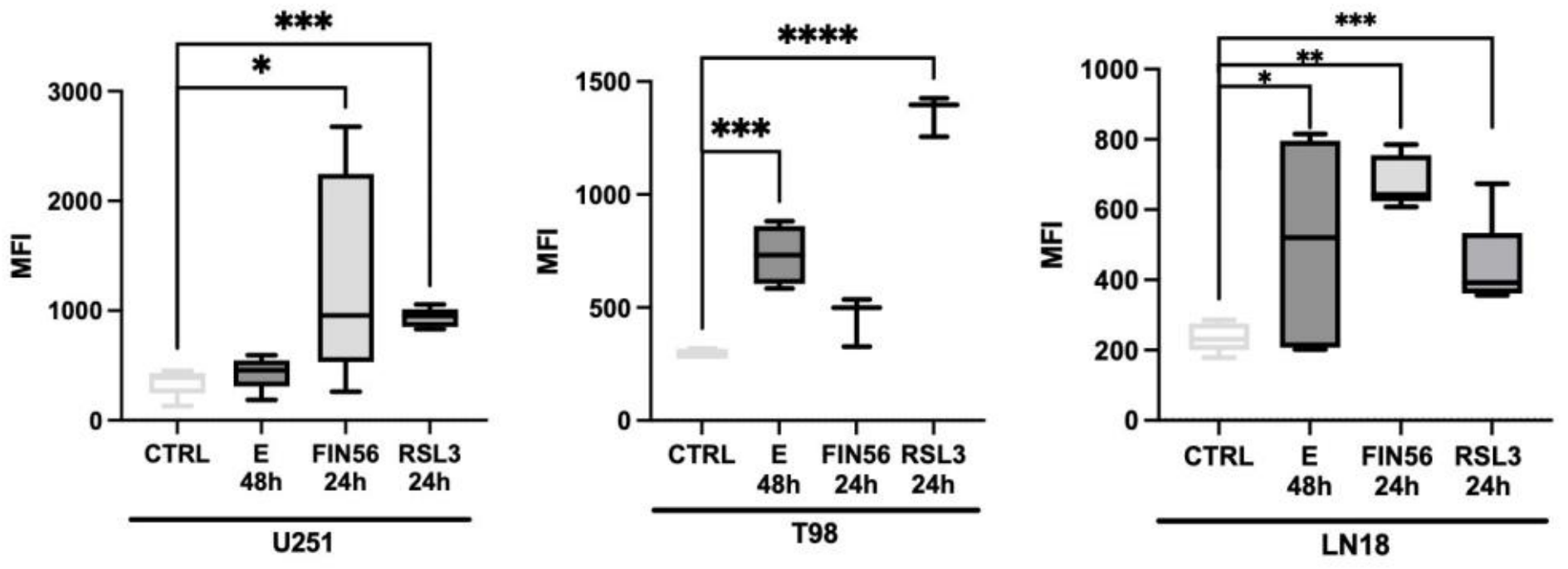

Supplementary 3. *Time-dependent ROS accumulation in drug-treated GBM cells*.
Flow cytometric analysis of mitochondrial reactive oxygen species (ROS) levels in resistant GBM cells after treatment with the selected drug at the endpoint. MitoSox was used as fluorescent probe to detect mitochondrial ROS. Results are expressed as mean fluorescence intensity (MFI) and normalized to control. Representative histograms and quantifications from three independent experiments are shown.

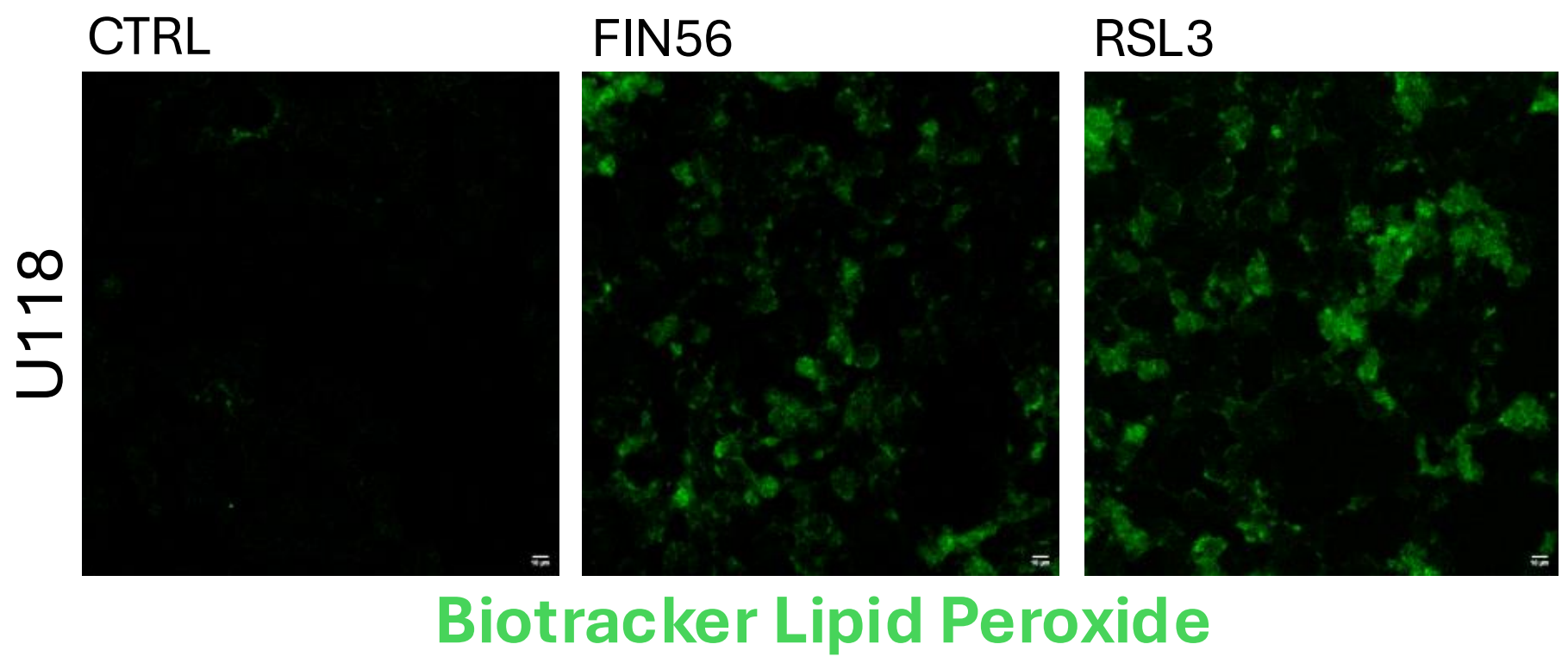

Supplementary 4. *Immunofluorescence detection of lipid peroxides in treated GBM cells.*
Immunofluorescence imaging was performed to visualize lipid-derived peroxide accumulation in GBM cells (U118) after drug exposure (FIN56 and RSL3). Cells were stained using BioTracker™ Green Lipid Peroxide Live Cell Ferroptosis Indicator and imaged under confocal microscopy. Representative images from three replicates are shown. Scale bar: 10 µm.

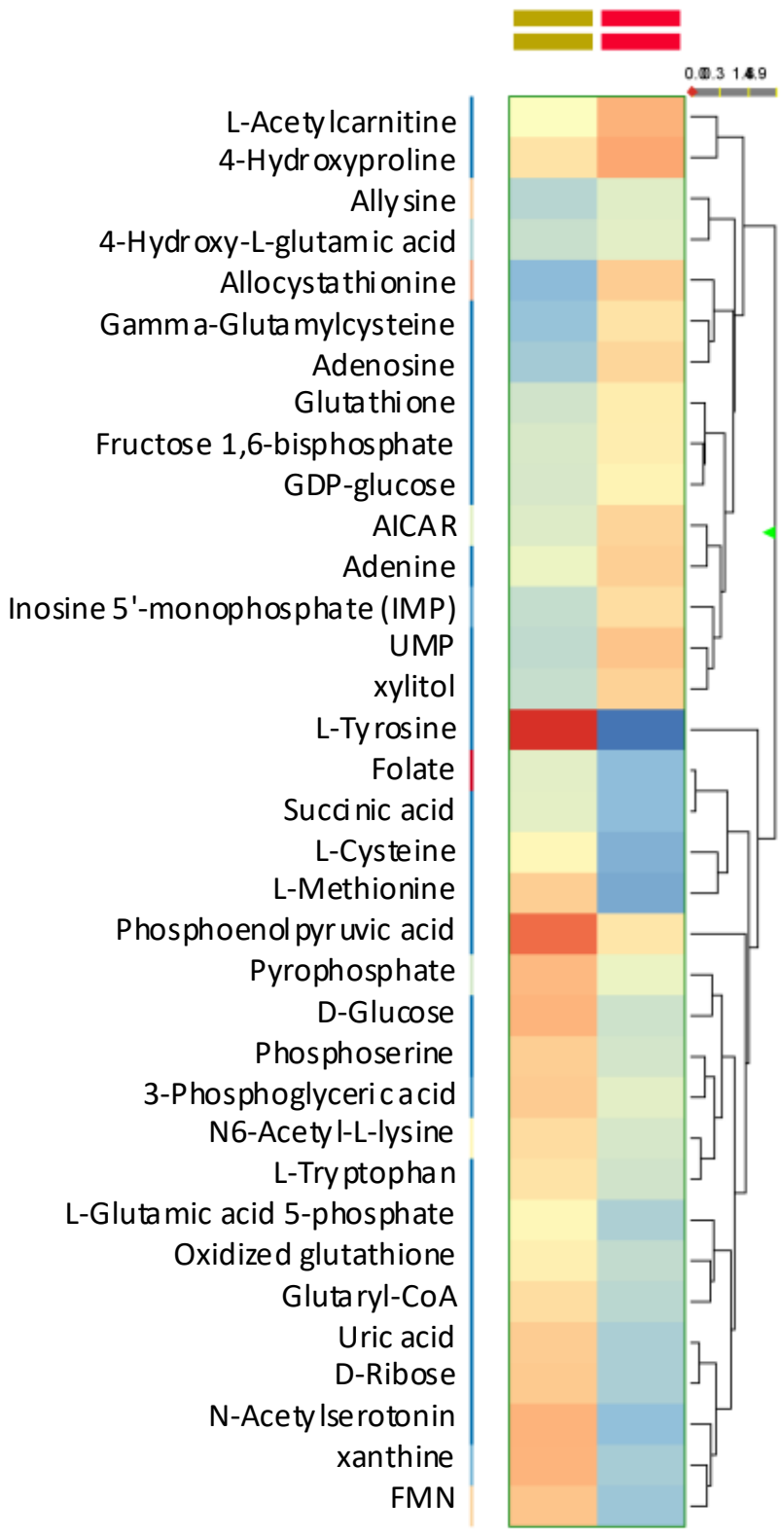

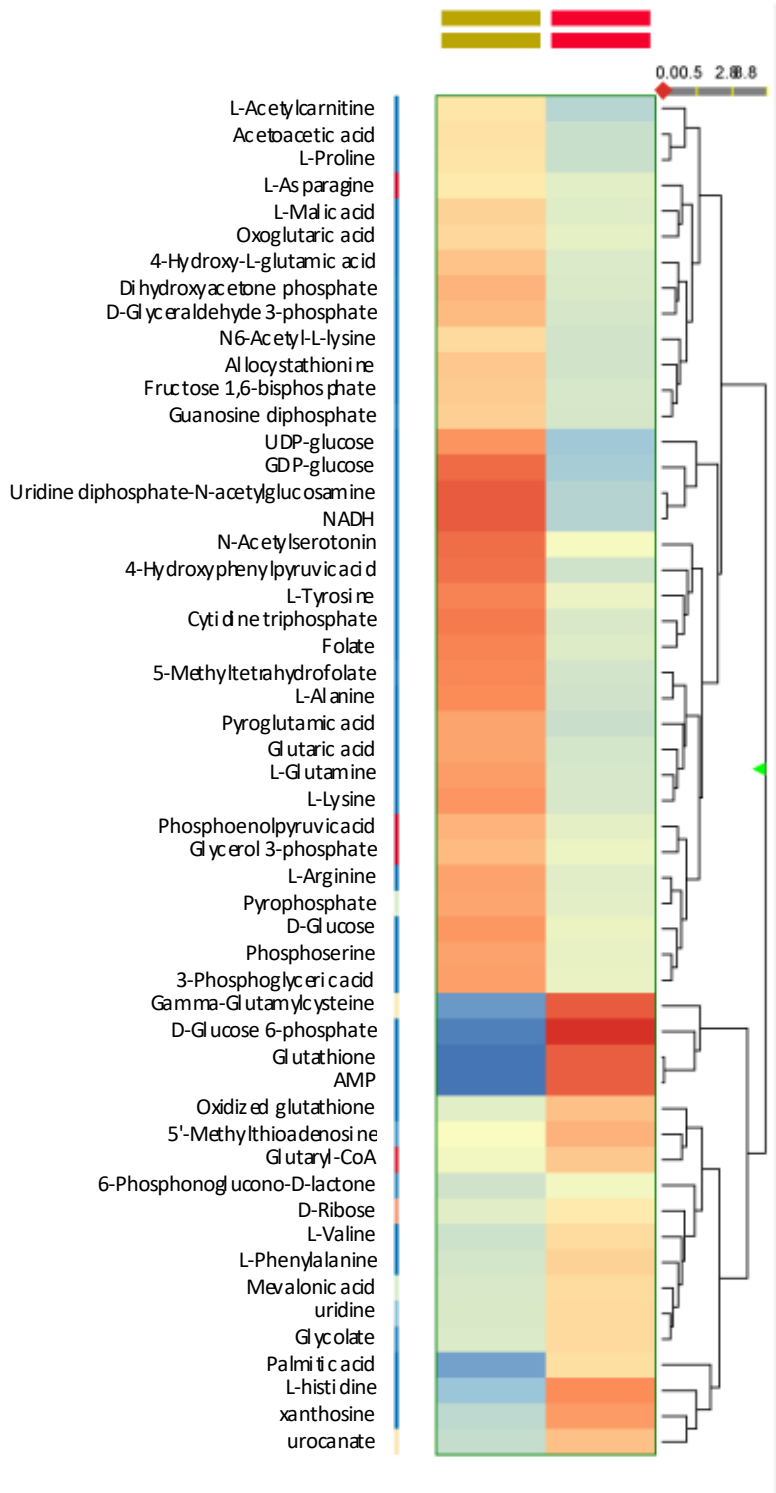

Supplementary 5. *Significant metabolites.*
List of statistically significant metabolites, including p-values, fold changes, and relative abundance values. The different Excel sheets correspond to the metabolites used for generating the heatmaps shown in Figure 4B.
